# Corneal surgery in keratoconus: which type, which technique, which outcomes?

**DOI:** 10.1186/s40662-016-0033-y

**Published:** 2016-01-18

**Authors:** Francisco Arnalich-Montiel, Jorge L. Alió del Barrio, Jorge L. Alió

**Affiliations:** IRYCIS. Ophthalmology Department, Ramón y Cajal University Hospital, Madrid, Spain; Cornea Unit, Hospital Vissum Madrid, Madrid, Spain; Cornea and External Diseases Service, Moorfields Eye Hospital, London, UK; Cornea, Cataract and Refractive Surgery Unit, Vissum Corporación, Alicante, Spain; Division of Ophthalmology, Universidad Miguel Hernández, Alicante, Spain

**Keywords:** Keratoconus, Deep anterior lamellar keratoplasty, Penetrating keratoplasty, Corneal transplant, Rejection

## Abstract

Keratoconus is a disease characterized by progressive thinning, bulging, and distortion of the cornea. Advanced cases usually present with loss of vision due to high irregular astigmatism. A majority of these cases require surgical intervention. This review provides an update on the current treatment modalities of corneal surgery available for the management of advanced corneal ectasias.

## Background

Corneal graft is the traditional recourse for advanced keratoconus [1]. There are many different grading schemes for keratoconus from scales based on outdated indices such as the Amsler-Krumeich scale, to scales using a variety of detailed metrics of corneal structure provided by anterior segment optical coherence tomography and Pentacam imaging. All these different scales do not always correlate well with disease impact. While there are eyes with milder disease that may exhibit contact lens intolerances, there are other eyes with severe disease that obtain good functional vision with contact lenses.

Therefore, although there is no precise definition for advanced disease, most specialists would agree that a keratoconus patient is eligible for corneal transplant when spectacle correction is insufficient, continued contact lens wear is intolerable, and visual acuity has fallen to unacceptable levels [2]. Nevertheless, there has been a strong push to extend other treatment modalities that were originally meant for mild to moderate disease such as ultraviolet crosslinking (UV-CXL) and intrastromal corneal ring segments (ICRS) to treat advanced disease. In 2014, Bowman Layer transplantation was also described for advanced keratoconus with extreme thinning/steepening [3]. These less troublesome therapeutic alternatives will seek to arrest disease progression, re-enable comfortable contact lens, or improve visual acuity to some extent, although rarely do the visual gains exceed one or two lines in advanced disease. These techniques would permit penetrating keratoplasty (PK) or deep anterior lamellar keratoplasty (DALK) to be postponed or avoided entirely [2].

In general, despite the excellent outcomes of PK, DALK may be preferred in patients with keratoconus because of the absence of risk of endothelial rejection, earlier tapering of steroids, decreased risk of secondary glaucoma, and increased wound strength [4]. The advantage of DALK is even more evident in patients with mental retardation in which PK has a higher incidence of postoperative complications such as globe rupture, corneal ulceration and graft rejection, as well as in phakic patients, and corneas with significant peripheral thinning [2].

PK would be considered more suitable in cases where endothelial dysfunction is present or when deep corneal scarring severely affects the visual axis up to the Descemet membrane (DM) level. It is not unusual for keratoconus to coexist with endothelial dysfunction; it might be underestimated as stromal thinning of keratoconus may mask the corneal edema. Fuchs endothelial dystrophy is the most common of such disorders, but also include posterior polymorphous dystrophy, a peculiar condition of endothelial depletion and guttae excrescences that may be the product of the keratoconus itself rather than a distinct entity [5]. If central deep corneal scarring is present, PK will provide a better visual acuity than DALK, but with a higher risk. In some instances, safety of DALK can outbalance the better visual acuity of PK. In fact, when corneal scars arise from previous hydrops, PK outcomes tend to be worse as the risk of graft rejection is higher [2]. In these cases, manual lamellar dissection for DALK is a good choice as Anwar’s big bubble technique is contraindicated owing to the high risk of perforation during surgery.

**Fig. 1:**
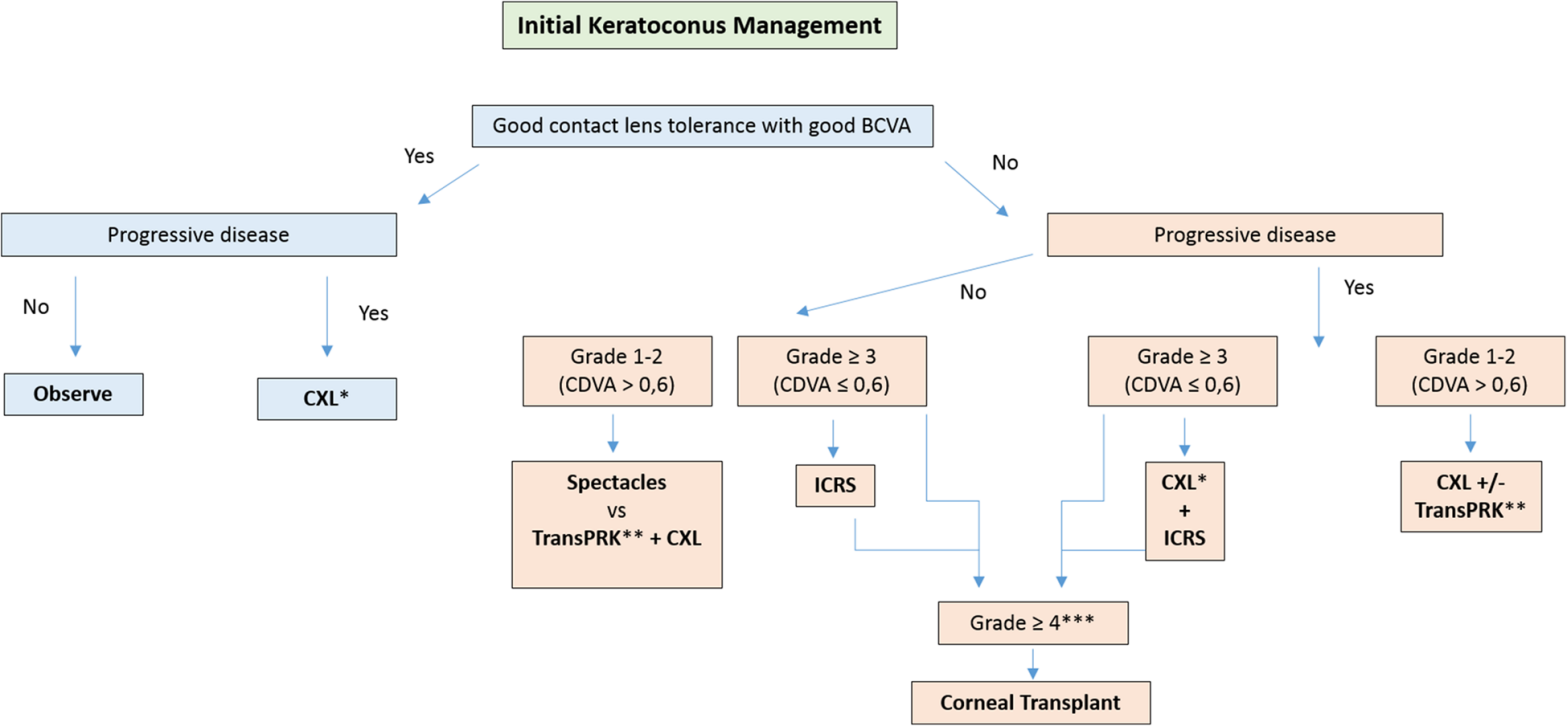
Decision tree for intervention at presentation in keratoconus. Grading according to the RETICS classification [1]. (* if thinnest point > 370 μm; ** wavefront guided transPRK (limited treatment) to reduce coma-like aberrations and increase CDVA; *** if corneal scarring, insufficient corneal thickness for ICRS implantation or ICRS failure with persistent contact/scleral lenses intolerance and poor CDVA)

While the scope of this article is mainly corneal grafting as treatment of keratoconus, it is important to point out that the main goal of treatment for keratoconus has changed over the last few years from that aiming to improve visual acuity with keratoplasty to a number of relatively new procedures focused on the prevention of disease progression or to restore/support contact lens tolerance by making wearing more comfortable. These include UV-CXL, ICRS, and a newly proposed type of “corneal transplant” known as Bowman Layer transplantation described by Gerrit Melles [3]. In Fig. 1, we present our decision tree for intervention at presentation in keratoconus.

## Review

A review of the literature on the topic of surgical treatment of keratoconus has received considerable attention and a formidable number and variety of surgical procedures even before keratoplasty was considered the most suitable procedure [6]. Surgical options that have been proposed include intraocular operations such as paracentesis of the anterior chamber, lens extraction or needling, or deviation of the pupil by incarcerating the iris in a corneal incision to achieve a stenopeic slit-like pupil; cone excision procedures; or flattening techniques by scar formation, brought by cauterization of the conus with chemicals, electrocautery, high frequency current or by splitting the DM [6].

Before keratoplasty became an option, Alfred Appelbaum in 1936 [7] stated concerning the surgical treatment of keratoconus, “Surgical intervention aims to produce flattening of the cornea in order to improve eyesight. When no degree of useful vision is obtained with the use of contact glasses, operative intervention may be considered – but no sooner. Only in cases of advanced or nearly hopeless conditions should the patient undergo operation. Most ophthalmologists agree with this. Too much cannot be expected of surgical treatment. At best, it gives a result far from ideal and none too lasting. The unsightliness which inevitably follows must be anticipated, and the appearance of the eye is always marred to some extent.”

Castroviejo, a Spanish ophthalmologist born in Logroño, Spain, performed the first PK for keratoconus in 1936 [6] in the Columbia Presbyterian Medical Center in New York. Several years later in an article about keratoplasty for the treatment of keratoconus, he concluded that keratoplasty was the only surgical procedure that fulfilled the two essential requirements for treating keratoconus: surgery had to be limited to the cornea, and the whole corneal protrusion had to be removed and replaced with normal tissue of normal curvature and thickness, leaving the pupillary area free of scarring. Based on his experience, when a suitable technique was used, the percentage of permanently, greatly improved vision increased from 75 % to 90 % [6].

Lamellar keratoplasty (LK) was described earlier than PK. Although Arthur von Hippel performed the first successful LK in man in 1888 [8] decades earlier than the first successful human PK by Edward Zinn, Von Hippel’s technique was abandoned in 1914 for PK and was not reintroduced until the 1940s [9]. However, the concept of deep LK extending down to DM is relatively new. Gasset reported a series of keratoconus patients in the late 1970s who received full-thickness grafts stripped of DM transplanted into relatively deep lamellar beds, and enjoyed good surgical results with 80 % of cases achieving 20/30 or better vision [10]. Dissection of host tissue ‘close to’ DM and the term ‘deep lamellar keratoplasty’ (DLK) in the conventional sense were first introduced by Archilla in 1984, who also showed the use of intrastromal air injection to opacify the cornea as a method to facilitate removal of host tissue [11]. Sugita and Kondo reported the first extensive study on the results of DLK compared with PK in 1997 [12]. They showed that postoperative visual acuity was similar between DLK and PK with no episodes of immunological rejection in over 100 eyes. Despite the clear benefits of DLK, the classical technique of removing stroma layer by layer was at that stage time-consuming and was greatly dependent on surgical experience. Only in the last two decades did DLK gain momentum thanks to improvement in surgical techniques and the availability of new surgical instruments and devices. The two most relevant papers on techniques were those from Melles and Anwar.

In 1999, Melles described a technique to visualize corneal thickness and dissection depth during surgery, which created an optical interface at the posterior corneal surface by filling the anterior chamber with air completely [13]. In 2002, Anwar described his popular “big-bubble” technique in baring DM by injecting air into the deep stroma to create a large bubble between the stroma and DM [14].

Approximately about 12–20 % of the keratoconus patients may require a corneal transplantation [15]. The Australian Graft Report of 2012 shows that keratoconus, with almost 1/3 of the corneal grafts performed, was the first reason for keratoplasty, followed by bullous keratoplasty and failed previous grafts. The 2012 Eye Banking statistical Report published by the Eye Banking Associations of America found that keratoconus was the reason for PK in 18 % of the cases and in 40 % of the DALK cases. Surprisingly, PK represented almost 80 % of the total grafts while DALK only accounted for 3 % of the total keratoplasties done, meaning that time-consuming and surgical experience is still a factor reducing the popularity of DALK in the US. Increasingly, however, DALK is becoming the preferred surgical option, largely thanks to improvements in operative technique, and now representing 10–20 % of all transplants for keratoconus and 30 % when eyes with previous hydrops are excluded [2]. In the UK, the percentage of transplants for keratoconus in which DALK was used increased from 10 % in 1999–2000 to 35 % in 2007–2008 [16].

## Penetrating keratoplasty in keratoconus

PK has traditionally been the surgery of choice for keratoconus, but nowadays lamellar techniques are the gold standard for patients with mild to moderate disease. Currently, an elective PK is reserved for those advanced cases where the DM and endothelium appear splitted due to a previous corneal hydrops. Frequently, a previous hydrops is not clearly reported by the patient, but in absence of an obvious endothelial split, deep stromal scars involving the DM are observed. In such cases a lamellar technique can still be attempted, mainly if these scars are not affecting the visual axis, but as the integrity of the DM is not intact any longer, this layer has a great tendency to rupture through the area of the scar (if and when a Big Bubble technique is used) and the surgery will need to be converted into a PK intraoperatively if a big tear is observed (longer than 2 to 3 clock hours).

PK technique for keratoconus does not differ significantly from the technique used for other etiologies, but some considerations should be taken into account:

1. Donor size:

A 7.5–8.5 mm host trephine (in relation with the corneal horizontal diameter) is often used and centered with the optical axis. However, the cone in keratoconus is often inferiorly displaced and should be fully removed to avoid residual or recurrent disease [17]. Therefore, the extent of the cone should be well understood before surgery and thinning mapped out by slit lamp examination, as this will be difficult to discern with the operating microscope. Fleischer iron ring formation, which usually circumscribes the cone, may assist on its delineation. Corneal topography is not reliable in advanced scarred conus and should not be considered for surgical planning. Donor size will then be adjusted in relation with the host limbal white-to-white measurement and conus extension, so grafts larger than 8.5 mm may occasionally be needed in severe conus, as well as partial decentration respecting the optical axis in cases of very advanced conus with a severe thinning up to the perilimbal area. Yet, the risk of rejection increases with grafts larger than 8.5 mm in diameter and when the graft-host junction moves closer to the limbus, both of these which should be considered during post-operative treatment and management [18, 19]. Decentered grafts can as well induce a significant irregular astigmatism into the visual axis that requires rigid lenses for visual rehabilitation of the patient and occasionally, a second centered graft for visual purposes.

The donor tissue trephine is routinely sized at 0.25 mm larger than the host trephine because, using current techniques, donor corneal tissue cut with a trephine from the endothelial surface measures approximately 0.25 mm less in diameter than host corneal tissue cut with the same diameter trephine from the epithelial surface [20]. Keratoconus patients may benefit from using same-diameter trephines for both donor and host tissues, which undersize the donor button and helps to reduce postoperative myopia [21, 22], but the surgeon should be aware that obtaining watertight wound closure with an undersized donor tissue can be challenging and may require additional sutures. Moreover, a flattened corneal contour could complicate contact lens fitting in the anisometropic patient. Laser excimer ablation for correction of a significant residual hyperopia after PK may also not be possible as it is not as predictable and efficient as it is with residual myopia, which will require phakic or pseudophakic piggyback intraocular lenses for patients who are intolerant of spectacles and contact lenses [23]. Considering the above, although undersizing the donor cornea may provide better visual outcome in patients with keratoconus, it should be selected carefully in PK. Axial length can be an important factor in the refractive error outcome following PK [24]. Ultrasound axial length measured from the anterior lens capsule to retina reveals a broad range in length from 18.77 to 25.65 mm. Reducing donor size, in a relatively short eye, could result in significant postoperative hyperopia, so same-size donor and host corneal buttons should not be used when the anterior lens-to-retina length is less than 20.19 mm, the mean length for non-keratoconic individuals with emmetropia.

2. Suturing technique:

Once the four cardinal 10-0 nylon sutures have been placed, the surgeon can use any of these preferred suture techniques: interrupted sutures (IS), combined continuous and interrupted sutures (CCIS), single continuous suture (SCS) or double continuous suture (DCS). IS should always be the closure method of choice in cases where a partial or complete suture removal in one region of the graft is likely to be necessary at some point during the postoperative period, examples include: pediatric keratoplasty (sutures becoming loose too quickly), vascularization in the host cornea (occasionally seen after a hydrops episode or contact lens related keratitis), multiple previous rejections or other inflammatory concomitant conditions that may predispose to localized vascularization, rejection, or ulceration of the donor tissue. Furthermore, large and decentered grafts that are placed close to the limbal area present an increased risk of rejection, thus making the use of IS necessary for its closure.

**Fig. 2:**
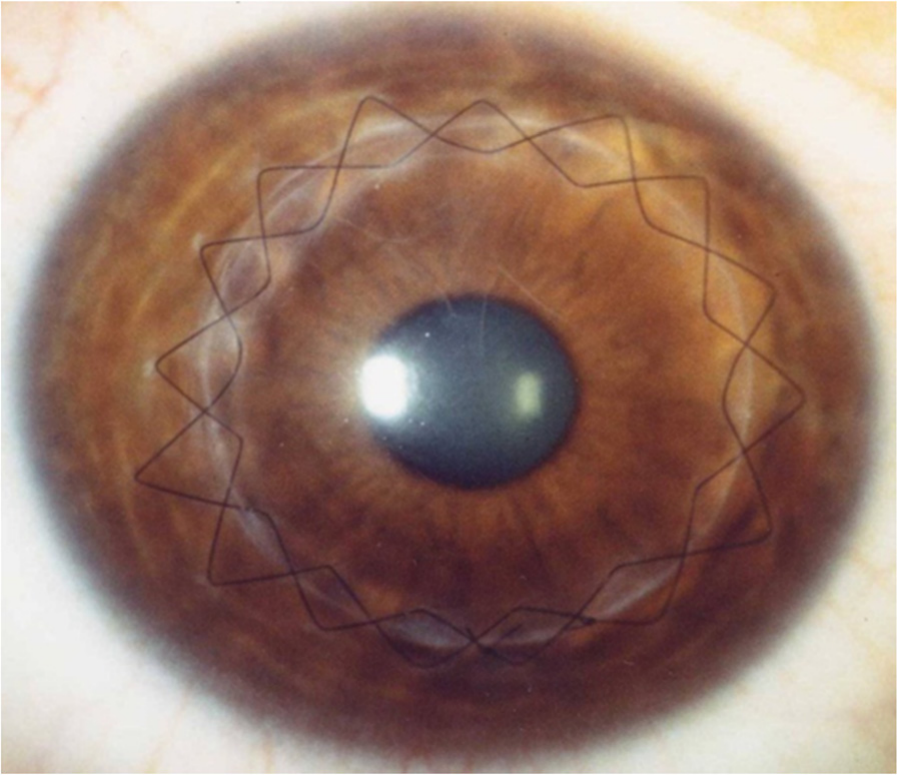
Slit-lamp image of a keratoconic eye after penetrating keratoplasty with a double continuous suture

However, most of the keratoconic eyes do not present any additional risk for graft rejection or infection, so a SCS or DCS is generally preferred by most surgeons. The advantages of a continuous suture are ease of placement, the ease with which the suture can be removed at a later date, and the potential for suture adjustment intrasurgically (with an intraoperative keratometer) and postoperatively to reduce astigmatism. With DCS, a 12-bite 10-0 nylon suture placed with bites at approximately 90 % depth and a second continuous suture (10-0 or 11-0 nylon) placed with bites alternating between each of the original suture’s bites for 360° at approximately 50–60 % corneal depth are used. The second suture is tied only with enough tension to take up slack in the suture. The second suture permits early removal or adjustment of the first 10-0 nylon suture for astigmatism control in 2–3 months; the second suture acts as a safety net if the deep suture breaks during the adjustment, and is generally left in place for 12–18 months postoperatively (Fig. 2).

IS, CCIS, and SCS have shown comparable postoperative astigmatism [25]. In addition, a comparison of astigmatism in keratoconus patients utilizing a single continuous versus a DCS showed that after suture removal, astigmatism between the two groups was comparable (DCS − 4.6 D, SCS − 5.2 D) [26]. Therefore, it is apparent that all methods of suture closure can work well. The ultimate choice rests with the surgeon.

**Fig. 3:**
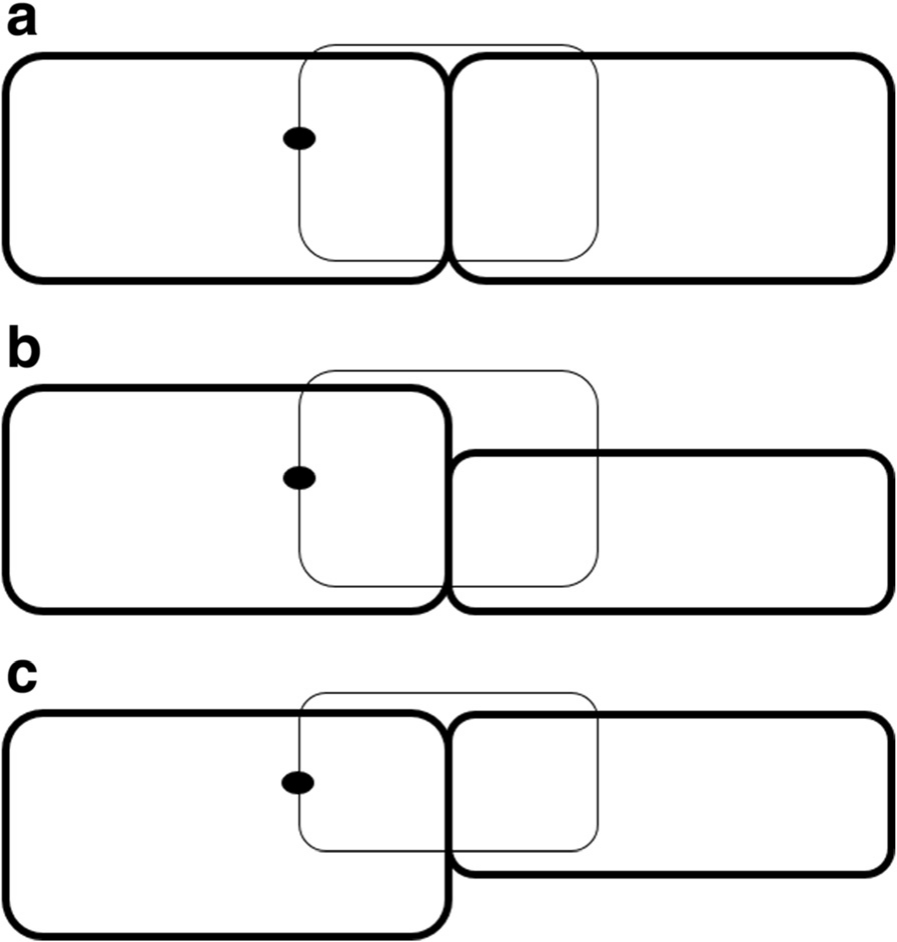
Graft-host junction alignment after suturing. Normal appearance of the graft-host junction with correct aligning of Bowman’s layer of the donor and host corneas, with needle passed at a 90 % depth on both sides (**a**). If care is not taken in cases of a thin recipient cornea, steps will remain at the graft-host junction, leaving an irregular astigmatism and exposed sutures that need to be replaced (**b**). To avoid this, a partial thickness bite (50–70 % depth) should be performed at the donor side (**c**)

Regardless of the preferred method, it is very important to have a clear concept of each suture technique. To give a basic idea for standard graft suturing, the needle is passed 90 % depth through the donor cornea and then through the host cornea. The ideal bite is as close to DM as possible, and there should be an equal amount of tissue purchased in the donor and host cornea in order to approximate Bowman’s layer in both the donor and host. Discrepancies frequently exist in the thickness of the donor and host corneas either when donor corneas are thick due to the hyperosmolar glycosaminoglycans in the preservation medium or fresh donor tissue is used in patients with severe corneal edema. This scenario is frequent in keratoconic eyes where the graft is sutured to a relatively thin host cornea. Closing Bowman’s layer to Bowman’s layer should always be attempted to avoid steps in the graft-host junction and subsequent exposed sutures. Therefore, in areas where the recipient cornea presents thin (assessed preoperatively by slit lamp examination) partial thickness bites (50–70 % depth) in the donor tissue should be in relation with deep bites (95 % depth) in the host thin stroma (Fig. 3).

The postoperative astigmatism management and elective suture adjustment/removal for PK in cases of previous keratoconus do not differ from other PK indications. A complete suture removal is generally recommended after 12–15 months.

3. Outcomes:

PK offers good long-term visual rehabilitation for keratoconus patients. Compared with other indications for PK, there is a relatively low rate of graft failure and long mean graft survival. Rejection rate has been reported to be 5.8–41 % with a long term follow-up with most rejections occurring in the first 2 years [27–31]. Larger host trephine size, male donor gender, and non-white donor race have been associated with increased rejection hazard [27]. Despite this observed rejection rate, only a 4–6.3 % graft failure rate has been reported with a mean follow-up of 15 years, and with an estimated 20 year probability of 12 % [27, 28, 32]. Fukoka et al. reported a cumulative probability of graft survival at 10, 20, and 25 years after PK of 98.8, 97.0 and 93.2 %, respectively, while Pramanik et al. estimated a graft survival rate of 85.4 % at 25 years after initial transplantation [28, 32]. Taken together, the existing evidence show that graft survival rate gradually decreases after 20 years post-PK.

An average best-corrected visual acuity (BCVA) in logarithm of the minimum angle of resolution (LogMAR) at preoperation, 10, 20, and 25 years after surgery of 1.54 ± 0.68, 0.06 ± 0.22, 0.03 ± 0.17, and 0.14 ± 0.42, respectively, have been reported [28]. Best spectacle-corrected visual acuity (BSCVA) of 0.14 ± 0.11 LogMAR has been reported with a mean period of 33.5 months, while a BSCVA of 20/40 or better with a mean follow-up of 14 years was observed in 73.2 % of patients [31, 32].

An open angle glaucoma rate of 5.4 % with a mean follow-up of 14 years has also been reported [32].

Claesson et al. reported a poorer survival and worse visual outcome of regrafts compared with first grafts in patients where the original indication was keratoconus: the failure rate was three times higher with regrafts and the observed visual acuity with preferred correction was ≥ 0.5 in 69 % of first grafts while only 55 % of regrafts achieved that level [33].

## Deep lamellar anterior keratoplasty in keratoconus

The goal of deep lamellar anterior keratoplasty in keratoconus is to achieve a depth of dissection as close as possible to DM. There are various ways to create a plane of separation between DM and the deep stromal layers mainly through variations of the two basic strategies: the Anwar big bubble method and the Melles manual dissection.

### Surgical techniques

1. The big bubble method

Anwar based the big bubble method on a discovery in 1998 that intrastromal injection of balanced salt solution (BSS) was often effective at establishing cleavage plane just above DM [34]. This takes advantage of the loose adhesion between DM and the posterior stroma. Anwar and Teichman later described the current big bubble procedure in 2002 using air instead of BSS [14].

**Fig. 4:**
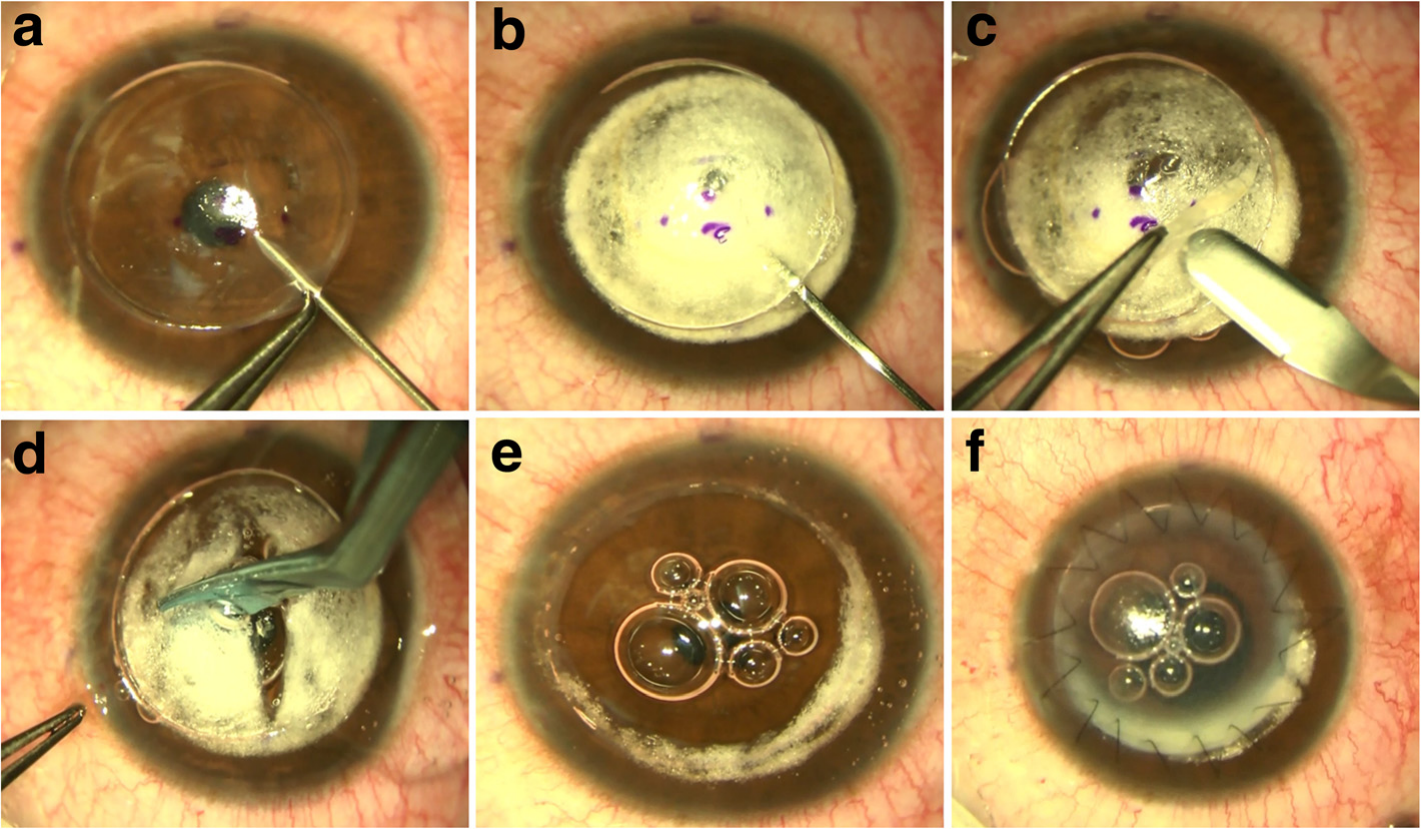
DALK Big Bubble Technique. After a partial trephination of 70–80 % of the corneal stroma 30 G needle (**a**). Once the air is injected, it produces a dome-shaped detachment of the DM that is seen under the surgical microscope as a ring meaning that the big bubble has been formed (**b**). A lamellar dissection with a Crescent blade of the anterior stroma is then performed (**c**) followed by the removal of the stromal tissue above the DM plane with spatula and scissors (**d**), making sure to first exchange the air in the supradescemetic plane with viscoelastic to avoid puncturing DM inadvertently. When all of the stromal tissue is successfully removed, the DM exposed is characteristically smooth (**e**), and the donor cornea without its DM and endothelium is then sutured with the preferred suture technique (**f**)

After a partial trephination of 70–80 % of the corneal stroma, pneumatic pressure is used to detach DM by injecting air into the deep stroma with a 30G needle. The air injected into the stroma produces a dome-shaped detachment of DM that is seen under the surgical microscope as a ring, which signifies that the big bubble has been formed. The stromal tissue above the DM plane is removed with spatula and scissors, making sure to first exchange the air in the supradescemetic plane with viscoelastic to avoid inadvertently puncturing the DM. When all of the stromal tissue is successfully removed, the DM exposed should be characteristically smooth (Fig. 4).

2. Melles manual method

**Fig. 5:**
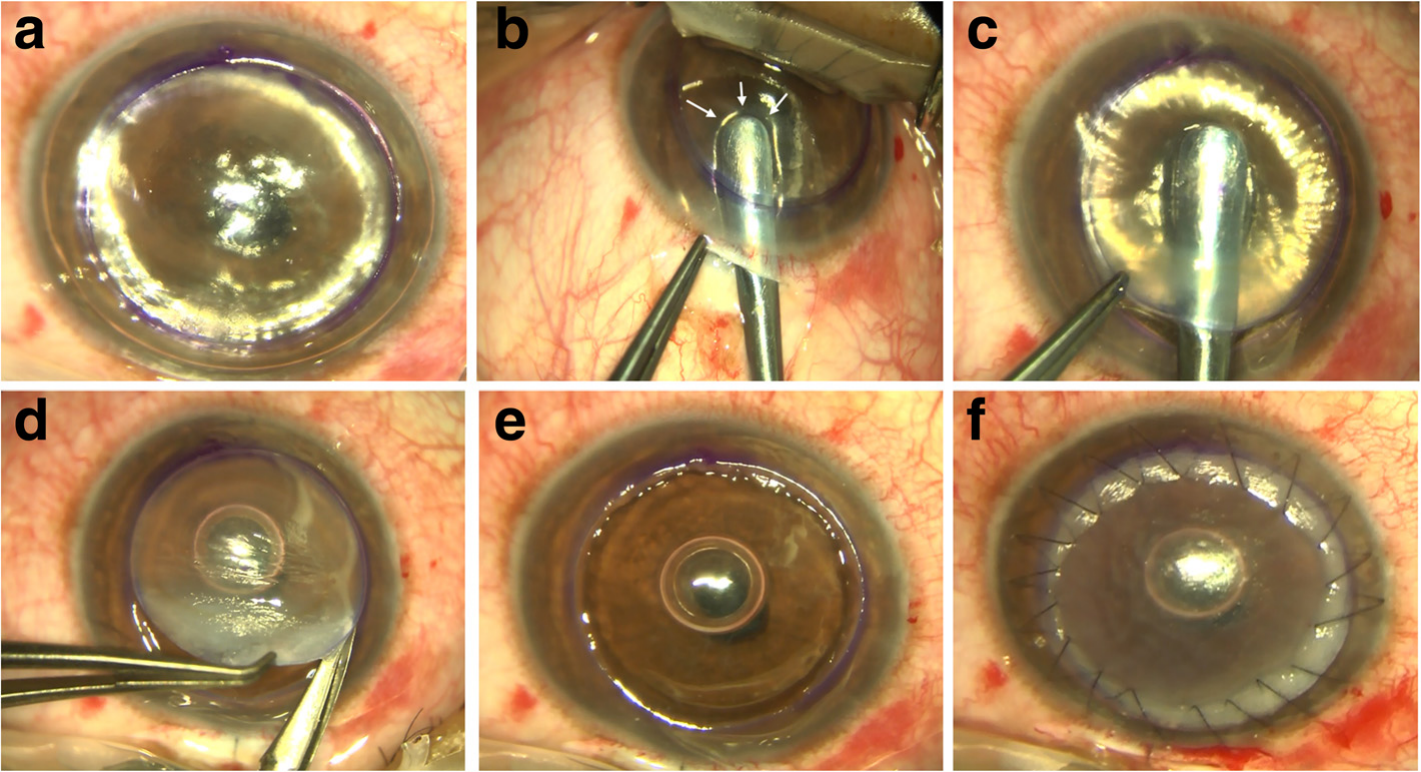
DALK Melles Technique. First, the anterior chamber is filled with air and a partial trephination of 70 % of the corneal stroma is performed (**a)**. Then, using a series of curved spatulas through a scleral pocket, the stroma is carefully dissected away from the underlying DM (**b)**. The difference in refractive index between air and corneal tissue creates a reflex of the surgical spatulas, and the distance between the instrument and reflex is used to judge the amount of remaining underlying tissue (*B*, *arrows*). Viscoelastic is injected through the scleral incision into the stromal pocket and the dissection can be completed through the trephination edge (**c**). Once completed, the superficial stroma is removed (**d)**, the DM exposed **e**, and the donor cornea sutured (**f**)

This technique is based on the air-endothelium interface [13]. First, the anterior chamber is filled with air. Then, using a series of curved spatulas through a scleral pocket, the stroma is carefully dissected away from the underlying DM. The difference in refractive index between air and corneal tissue creates a reflex of the surgical spatulas, and the distance between the instrument and reflex is used to judge the amount of remaining cornea. Viscoelastic is injected through the scleral incision into the stromal pocket. Once the desired plane is reached, the superficial stroma is removed using trephine and lamellar dissection (Fig. 5).

Over the years, there have been many variations to the standard technique. Lamellar dissection can be made with a diamond knife, nylon wire, microkeratome [35] or femtosecond laser. To help in guiding the dissection plane, trypan blue, ultrasound pachymetry [36] or real time optical coherence tomography [37] (OCT) has been used. Partharsathy et al. describe a “small bubble” technique for confirming the presence of the big bubble [38].

For corneas with extreme peripheral thinning, a modified procedure has been proposed dubbed “tuck-in lamellar keratoplasty” [39, 40]. In this technique, the central anterior stromal disc is removed and a centrifugal lamellar dissection is performed using a knife to create a peripheral intrastromal pocket extending 0.5 mm beyond the limbus. The donor cornea is prepared in such a way that it has a central full thickness graft with a peripheral partial thickness flange. The edges of a large anterior lamellar graft are tucked in below to add extra thickness.

### Outcomes

Most studies have found equivalent visual and refractive results between PK and DALK, although 20/20 vision seems more likely after PK [16, 41], provided that stromal dissection reaches the level or close to the DM [16, 41–46]. For instance, in a recent study consisting Australian patients, which included 73 consecutive patients with keratoconus, the mean BCVA was not significantly different for DALK (0.14 logMAR, SD 0.2) versus PK (0.05 logMAR, SD 0.11) [16, 41]. A review of published literature that included 11 comparative studies on DALK and PK found that visual and refractive outcomes were comparable if the residual bed thickness in DALK cases were between 25 and 65 μm [4].

In studies where the visual outcomes of DALK where inferior to PK [47], the dissection plane was “pre-descemetic” and the incomplete stromal dissection and the not fully baring of the DM had a negative impact on the results [47]. The problem seems to be related to the depth of the undissected stromal bed rather than to its smoothness as pre-descemetic DALKs performed by laser ablation did not outperform those dissected manually.

The recently published Australian graft registry data compared the outcomes of PKs and DALKs performed for keratoconus over the same period of time and found that overall, both graft survival and visual outcomes were superior for PK. In a recent study from the UK, Jones et al. compared the outcomes after PK and DALK for keratoconus [16]. The risk of graft failure for DALK was almost twice that for PK. In day-to-day clinical practice, visual outcomes with DALK although comparable with PK, may be slightly inferior or less predictable compared with PK, given surgical inexperience, and unpredictable issues with respect to residual stromal thickness and DM folds. Nonetheless, elimination of risk of endothelial rejection compensates for this difference.

Lastly, one of the important advantages of DALK is a lower rate of endothelial loss compared with PK. The reported endothelial cell loss is as high as 34.6 % after PK, whereas for after DALK, cell loss was only 13.9 % [48].

### Use of femtosecond laser in corneal graft for keratoconus

**Fig. 6:**
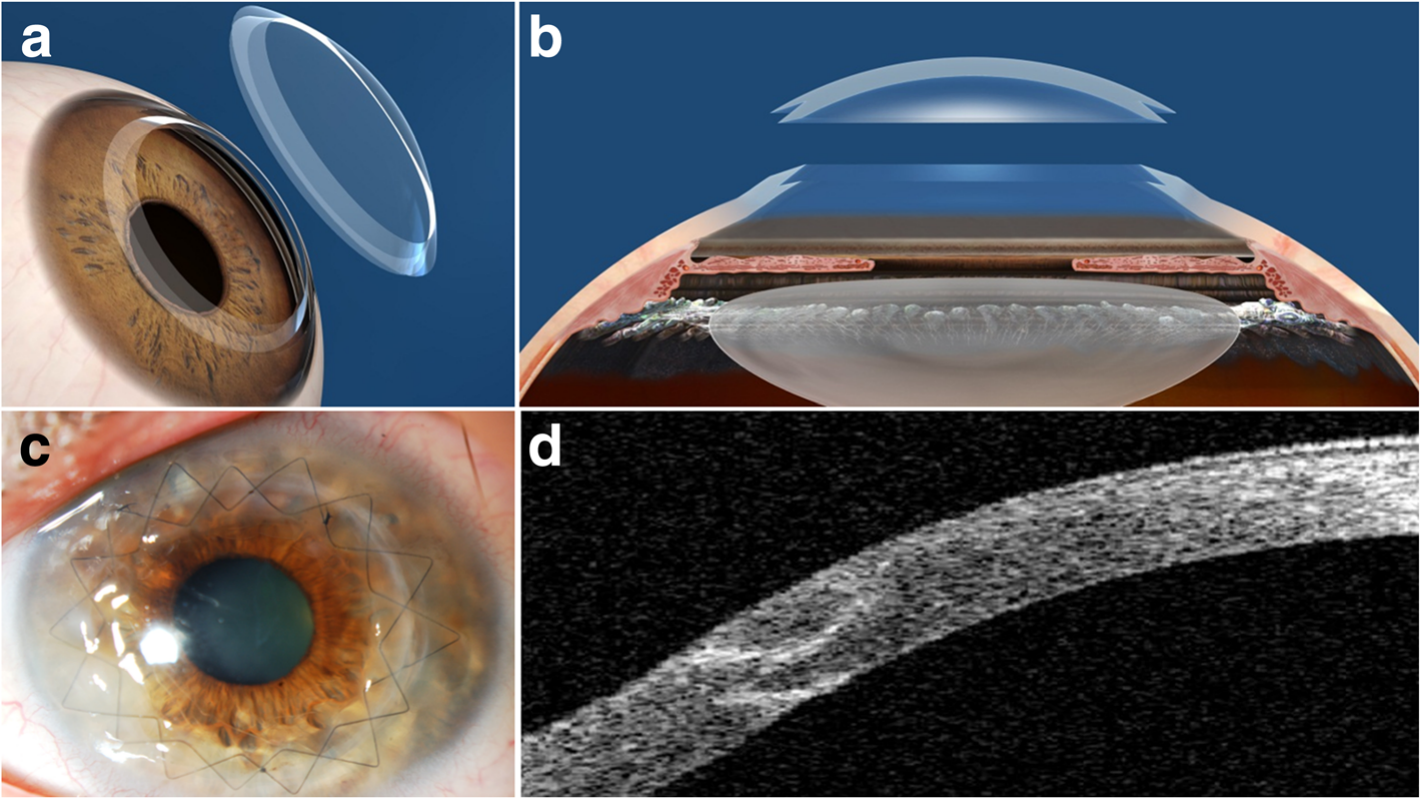
Femtosecond laser assisted penetrating keratoplasty with a “Zig-Zag” edge profile (**a**, **b**: courtesy of Abbott Medical Optics, USA). Postoperative clinical picture (**c**) and an anterior segment OCT capture (**d**) where it is possible to appreciate the zig-zag edge profile at the host-donor interface with a perfect coalescence of the edges

In the last decade, the femtosecond laser is one of the most important innovations in corneal transplant surgery for keratoconus. The laser allows the surgeon to focus the laser energy at a particular depth and then rapidly cut the tissue at that depth without causing any additional injury to the surrounding tissue. This permits doing lamellar dissection with high precision and also allows the surgeon to pattern these cuts into shapes (often referred to as mushroom or zig zag) creating a highly precise incision resulting in a perfect match of the donor tissue and the host tissue and a stronger junction and quicker visual recovery [49] (Fig. 6).

### Complications

Allograft reactions are less frequent in DALK than in PK and less likely to result in graft failure if correct treatment is administered. Subepithelial and stromal rejection after DALK has been reported to be in the range of 3–14.3 % whereas in PK, it ranges from 13 to 31 % in the first 3 years after surgery [2]. Endothelial rejection is not an issue in DALK.

Increases in intraocular pressure (IOP) following DALK has been reported in 1.3 % of operated eyes, compared with 42 % of eyes after PK [48]. Development of glaucoma may also be up to 40 % less than PK [50]; it is attributed to the lower steroid requirement of DALK [51].

Urretz-Zavalia Syndrome was first reported following PK in keratoconus. It causes fixed, dilated pupil with iris atrophy that is a rare entity following DALK [52].

There are also a few complications that are unique to DALK and the presence of a donor-host interface. One of the major problems with DALKs is intraoperative DM perforation, which may occur in 0–50 % of the eyes [2], which has also been described to occur weeks after an uneventful surgery [53]. Surgeon’s inexperience, corneal scarring near the DM, and advanced ectasias with corneal thickness less than 250 μm increase this risk [54, 55]. Depending on the size of the perforation, conversion to PK may be required to avoid double anterior chamber and persistent corneal edema, especially when the rupture leads to the collapse of the anterior chamber (macroperforation). Incidence of pseudo anterior chamber or double anterior chamber is in the range of 1 % [56]. It can occur because of retention of fluid secondary to breaks in the DM or because of incomplete removal of viscoelastic in the interface [57]. Large pseudo chambers must be managed surgically by drainage of the fluid and anterior chamber injection of air or gas [58], while small pseudo chambers normally end up resolving spontaneously [59]. The presence of DM folds caused by a mismatch between donor button and the recipient bed is usually transient and would disappear over time, but interface wrinkling when central and persistent may affect quality of vision [60]. Occasionally, an eye with an anatomically correct DALK may require a secondary reoperation to interface haze and poor visual acuity, usually stemming from incomplete or pre-descemetic stromal dissection [2]. Interface keratitis is a serious complication of DALK and its caused mainly by Candida [61], but Klebsiella pneumonia [62] and nontuberculous mycobacteria [63] have also been isolated in several cases. Conservative treatment is usually unsuccessful and most cases need a therapeutic PK [61]. Interface vascularization can occur because of inflammatory, infective, and traumatic episodes which can be treated with bevacizumab injection [64].

## Keratoconus recurrence after corneal transplantation

**Fig. 7:**
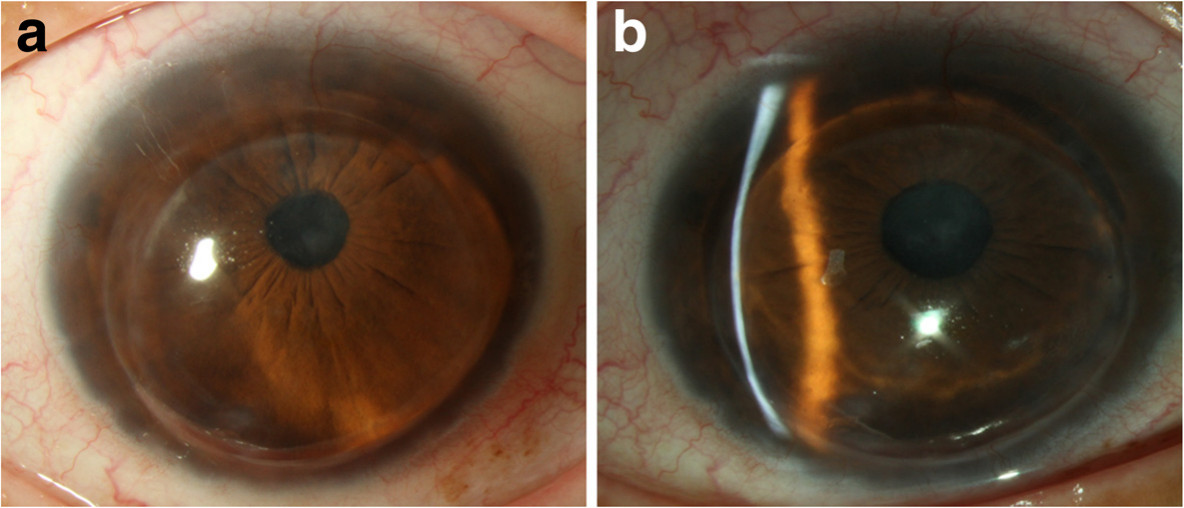
Keratoconus recurrence. Slit lamp image of the recurrence 17 years after a penetrating keratoplasty (**a**). Observe the severe thinning of the recipient stroma at the graft-host junction (**b**)

We have already discussed the beneficial long-term results of the different options of corneal grafting for keratoconus. de Toledo et al. observed a progressive increase of keratometric astigmatism in 70 % of their cases from 10 years after suture removal, following an initial phase of refractive stability during the first 7 years after PK for keratoconus (4.05 ± 2.29 D 1 year after suture removal, 3.90 ± 2.28 D at year 3, 4.03 ± 2.49 D at year 5, 4.39 ± 2.48 D at year 7, 5.48 ± 3.11 D at year 10, 6.43 ± 4.11 D at year 15, 7.28 ± 4.21 D at year 20, and 7.25 ± 4.27 D at year 25), suggesting that a late recurrence of the disease may occur with an increasing risk over time [17]. Actually, a 20 year post-PK probability of 10 % have been reported previously, with a mean time to recurrence of 17.9–21.9 years. Given the younger age at which keratoconus patients undergo corneal transplantation, these long-term findings should be explained to patients and incorporated into preoperative counselling [27, 32, 65] (Fig. 7).

It is well known how other corneal stromal dystrophies such as granular or lattice dystrophy tend to recur into the donor cornea due to either colonization of the new stroma by the abnormal host keratocytes or epithelial secretion in the early stages. In keratoconus, this host keratocyte invasion has not been well proven to be the main etiology for the post graft recurrent ectasia, but is likely to be related to the early keratoconic changes observed in the histology of explanted donor buttons after regrafting [65–67]. Post-graft ectasia is often preceded by thinning of the recipient stroma at the graft-host junction, so disease progression at the host stroma is likely to be the underlying reason for these cases of recurrent ectasia and progressive astigmatism [17, 65]. In such cases, a mean keratometric sphere and cylinder increase of 4D and 3D, respectively, between final suture removal and diagnosis can be observed [65].

The management of recurrent ectasia after corneal grafting should be spectacle adjustment if low astigmatism levels are induced, and rigid/hybrid gas permeable contact lenses with higher levels of astigmatism or significant anisometropia. For more advanced cases, scleral lenses may be considered before a surgical approach. If a second corneal transplant is required, either a new full thickness PK versus LK can be considered. Large grafts are usually necessary as the whole area of thinning should be included within the graft limits in order to excise the whole cone to avoid a new recurrence and also to avoid suturing through a thin recipient cornea. As large grafts are associated with increased risk of rejection and glaucoma, lamellar techniques by manual dissection of the host and donor corneal stroma are always preferable as far as the donor endothelium presents healthy without signs of failure. If femtosecond dissection of the lamellar bed is chosen, gentian violet and cyanoacrylate glue can be used in the area of thinning as masking agents to minimize the risk of perforation [68]. Limbus may have to be recessed while suturing very large grafts that sit close to the limbus in order to avoid passing the suture through the host’s conjunctiva. Recurrence after regrafting has also been reported, so much so that it may require a third graft for visual rehabilitation [65].

Keratoconus recurrence after DALK has not been described. Very little evidence about its real incidence and impact is currently available. Feizi et al. reported a case where keratoconus recurred only 49 months after DALK [69]. They suggested that the time interval from transplantation to recurrence may be shorter after DALK than after PK, but this has not been supported or confirmed by other authors [70]. Further studies analyzing the long term outcomes after DALK for keratoconus is required in order to assess its impact [68].

## A glance into the future

Keratoconus is a corneal disease that primarily affects the corneal stroma and Bowman’s layer. Current research and future therapeutic directions are focusing on the regeneration of corneal stroma by little to no invasive procedures to avoid the common complications that we still see even with LK techniques.

In the last few years, various studies have shown that CXL may offer some promise in slowing the progression of the disease [71, 72]. New modalities of CXL are being explored to improve the outcomes. CXL along with topography-guided photorefractive keratectomy (PRK) in order to provide better visual rehabilitation in patients with keratoconus is already being used [73]. A novel approach to enhance riboflavin penetration is based on iontophoresis, a non-invasive system aimed to enhance the delivery of charged molecules into tissues using a small electric current. It has been shown that an iontophoresis imbibition lasting 5 min achieves a sufficient riboflavin concentration in the corneal stroma for CXL treatment, with the advantage of shortening the imbibition time while preserving epithelial integrity [74]. Accelerated CXL was introduced in clinical practice in order to shorten the time required for a CXL procedure [75]. This technique is based on the Bunsen-Roscoe law of photochemical reciprocity. That is, the same photochemical effect can be achieved with reducing the irradiation interval provided that the total energy level is kept constant by a corresponding increase in irradiation intensity. In this modality, pulsed accelerated corneal collagen crosslinking seems to be more effective than continuous light accelerated corneal collagen crosslinking [76].

Melles at al. recently described a new technique where an isolated Bowman’s layer is transplanted into a mid-stromal manually dissected corneal pocket in patients with an advanced (Stage III-IV) keratoconus [77]. They observed a modest improvement in the maximum keratometry and BSCVA, but an unchanged best contact lens corrected visual acuity (BCLVA). This is a new and interesting approach that could have its indication for those advanced keratoconus unsuitable for corneal collagen crosslinking or intracorneal ring segments and intolerant to contact lenses, but without visually significant corneal scars and therefore good BCLVA. In such cases, Bowman’s transplant could avoid or postpone the necessity of keratoplasty if the mild observed corneal flattening enables continued contact lens wear and the cone is stabilized (as it has been reported to happen, but only with a sample of 20 eyes and a short mean follow-up of 21 months). Further research by alternative authors with a larger sample and longer follow up is needed before introducing this technique into routine clinical practice.

As discussed, Bowman’s transplantation could have some benefits in cases of advanced keratoconus, but even if these results are finally confirmed by other authors, they offer a mild improvement to these patients without a significant functional/anatomical rehabilitation. Thus, further techniques may focus on attempting the subtotal regeneration or substitution of the corneal stroma in order to achieve better results. Different types of stem cells have been used in various ways by several research groups in order to find the optimal procedure to regenerate the human corneal stroma: Corneal Stromal Stem Cells (CSSC), Bone Marrow Mesenchymal Stem Cells (BM-MSCs), Adipose Derived Adult Mesenchymal Stem Cells (ADASCs), Umbilical Cord Mesenchymal Stem Cells (UCMSCs), and Embryonic Stem Cells (ESCs) [78]. These approaches can be classified into four techniques:

A. Intrastromal injection of stem cells alone:

Direct injection of stem cells inside the corneal stroma has been assayed in vivo in some studies, demonstrating the differentiation of the stem cells into adult keratocytes without signs of immune rejection. Our group showed the production of human extracellular matrix (ECM) when human ADASCs (h-ADASC) were transplanted inside the rabbit cornea [79]. Du et al. reported a restoration of the corneal transparency and thickness in lumican null mice (thin corneas, haze and disruption of normal stromal organization) 3 months after the intrastromal transplant of human CSSCs. They also confirmed that human keratan sulphate was deposited in the mouse stroma and the host collagen lamellae were reorganized, which led to the conclusion that delivery of h-CSSCs to scarred human stroma may alleviate corneal scars without requiring surgery [80]. Very similar findings were reported by Liu et al. using human umbilical mesenchymal stem cells (UMSCs) in the same animal model [81]. Recently, Thomas et al. found that in a mice model for mucopolysaccharidosis, transplanted human UMSC participate both in extracellular glycosaminoglycans (GAG) turnover and enable host keratocytes to catabolize accumulated GAG products [82]. In our experience, the production of human ECM by implanted mesenchymal stem cells occurs, but not quantitatively enough to be able to restore the thickness of a diseased human cornea. However, the direct injection of stem cells may provide a promising treatment for corneal dystrophies including keratoconus, via the regulation of abnormal host keratocyte collagen production to enable collagen microstructure reorganization and corneal scarring modulation.

B) Intrastromal implantation of stem cells together with a biodegradable scaffold:

In order to enhance the growth and development of the stem cells injected into the corneal stroma, transplantation with biodegradable synthetic extracellular matrixes (ECMs) has been performed. Espandar et al. injected h-ADASCs with a semisolid hyaluronic acid hydrogel into the rabbit corneal stroma. They report better survival and keratocyte differentiation of the h-ADASCs when compared with injection alone [83]. Ma et al. used rabbit adipose-derived stem cells (ADSCs) with a polylactic-coglycolic (PLGA) biodegradable scaffold in a rabbit model of stromal injury and observed newly formed tissue with successful collagen remodeling and less stromal scarring [84]. Initial data show that these scaffolds could enhance stem cell effects over corneal stroma, although more research is required.

C) Intrastromal implantation of stem cells with a non-biodegradable scaffold:

At the present moment, no clinically viable human corneal equivalents have been produced by tissue engineering methods. The major obstacle to the production of a successfully engineered cornea is the difficulty with reproducing (or at least simulating) the stromal architecture. The majority of stromal analogs for tissue engineered corneas have been created by seeding human corneal stromal cells into collagen-based scaffoldings, which are apparently designed to be remodeled (see Ruberti et al. 2008 for a general review of corneal tissue engineering) [85]. The major drawback of these analogs is their lack of strength, thus unable to restore the normal mechanical properties of the cornea. New and improved biomaterials compatible with human corneas and with enhanced structural support have been developed leading to advanced scaffolds that can be used to engineer an artificial cornea (keratoprosthesis) [78]. The combination of these scaffolds with cells can generate promising corneal stroma equivalents, and some studies have already been published that use mainly corneal cell lines providing positive results regarding adhesion and cellular survival in vitro [86]. Our opinion is that stem cells do not differentiate properly into keratocytes in the presence of these synthetic biomaterials. Doing so makes them lose their potential benefits and not resolve the major drawbacks with such substitutes: their relatively high extrusion rate and lack of complete transparency [87].

**Fig. 8:**
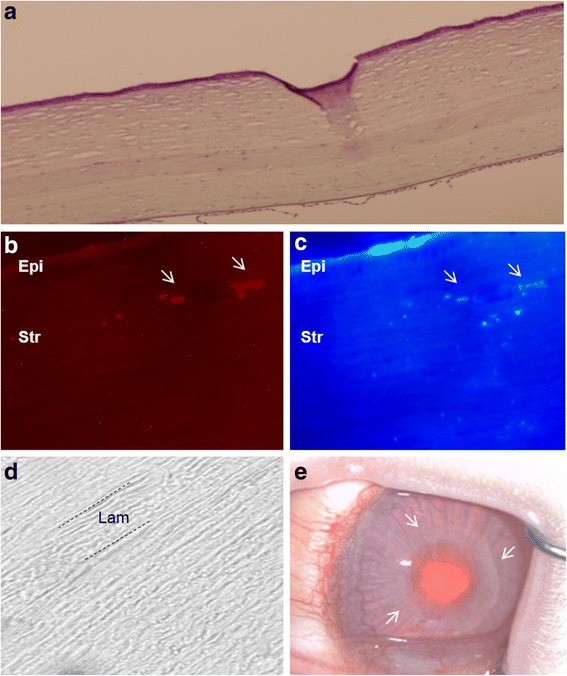
Reconstruction of corneal stroma. **a**: Hematoxylin-eosin staining of a rabbit cornea with an implanted graft of decellularized human corneal stroma with h-ADASC colonization: hypocellular band of ECM without vessels or any inflammatory sign (magnification 200X); **b**: Human cells labeled with CM-DiI around and inside the implant that express (**c**) human keratocan (human adult keratocyte specific marker; magnification 400X), confirming the presence of living human cells inside the corneal stroma and their differentiation into human keratocytes (*arrows*); **d**: Phase-contrast photomicrographs showing a morphologically unaltered corneal stroma (magnification 400X); **e**: The graft remains totally transparent after 12 weeks of follow-up (magnification 2X) (arrows point to the slightly visible edge of the graft). Abbreviations: Epi: epithelium; Str: stroma; Lam: Lamina

D) Intrastromal implantation of stem cells with a decellularized corneal stromal scaffold:

The complex structure of the corneal stroma has not yet been replicated, and there are well-known drawbacks to the use of synthetic scaffold-based designs. Recently, several corneal decellularization techniques have been described, which provide an acellular corneal ECM [88]. These scaffolds have gained attention in the last few years as they provide a more natural environment for the growth and differentiation of cells when compared with synthetic scaffolds. In addition, components of the ECM are generally conserved among species and are tolerated well even by xenogeneic recipients. Keratocytes are essential for remodeling the corneal stroma and for normal epithelial physiology [89]. This highlights the importance of transplanting a cellular substitute together with the structural support (acellular ECM) to undertake these critical functions in corneal homeostasis. To the best of our knowledge, all attempts to repopulate decellularized corneal scaffolds have used corneal cells [90–92], but these cells have major drawbacks that preclude their autologous use in clinical practice (damage of the donor tissue, lack of cells and inefficient cell subcultures), thus the efforts to find an extraocular source of autologous cells. In a recent study by our group, we showed the perfect biointegration of human decellularized corneal stromal sheets (100 μm thickness) with and without h-ADASC colonization inside the rabbit cornea in vivo (Fig. 8a and b), without observing any rejection response despite the graft being xenogeneic [93]. We also demonstrated the differentiation of h-ADASCs into functional keratocytes inside these implants in vivo, which then achieved their proper biofunctionalization (Fig. 8c). In our opinion, the transplant of stem cells together with decellularized corneal ECM would be the best technique to effectively restore the thickness of a diseased human cornea such as that in keratoconus. Through this technique and using extraocular mesenchymal stem cells from patients, it is possible to transform allergenic grafts into functional autologous grafts, theoretically avoiding the risk of rejection.

## Conclusion

Treatment of keratoconus has experienced great advances in the last two decades. From being limited only to rigid gas permeable contact lens wear and PK for the most advanced cases, to having different therapeutic alternatives currently to treat not only the cone and postpone/avoid the necessity of a corneal transplant, but also being able to halt the progression of the disease with a very high rate of efficacy and safety. Also, the advances in refractive surgery including surface corneal ablation treatments and phakic intraocular lenses have allowed a better management and visual rehabilitation of these patients after a corneal transplant is required, being able to achieve, in many cases, a 20/20 unaided vision. The future expected advances in transepithelial crosslinking, nanotechnology, and regenerative medicine predicts an exciting future in this field and we will be looking forward to updating these guidelines.
